# HNF4A modulates glucocorticoid action in the liver

**DOI:** 10.1016/j.celrep.2022.110697

**Published:** 2022-04-19

**Authors:** A. Louise Hunter, Toryn M. Poolman, Donghwan Kim, Frank J. Gonzalez, David A. Bechtold, Andrew S.I. Loudon, Mudassar Iqbal, David W. Ray

**Affiliations:** 1 Faculty of Biology, Medicine and Health, University of Manchester, Manchester M13 9PT, UK; 2 Oxford Centre for Diabetes, Endocrinology and Metabolism, University of Oxford, Oxford OX3 7LE, UK; 3 Laboratory of Metabolism, Center for Cancer Research, National Cancer Institute, National Institutes of Health, Bethesda, MD 20892, USA; 4 NIHR Oxford Biomedical Research Centre, John Radcliffe Hospital, Oxford OX3 9DU, UK

**Keywords:** glucocorticoid receptor, nuclear receptor, HNF4A, liver, ChIP, chromatin, tissue specificity, mouse, cistrome

## Abstract

The glucocorticoid receptor (GR) is a nuclear receptor critical to the regulation of energy metabolism and inflammation. The actions of GR are dependent on cell type and context. Here, we demonstrate the role of liver lineage-determining factor hepatocyte nuclear factor 4A (HNF4A) in defining liver specificity of GR action. In mouse liver, the HNF4A motif lies adjacent to the glucocorticoid response element (GRE) at GR binding sites within regions of open chromatin. In the absence of HNF4A, the liver GR cistrome is remodeled, with loss and gain of GR recruitment evident. Loss of chromatin accessibility at HNF4A-marked sites associates with loss of GR binding at weak GRE motifs. GR binding and chromatin accessibility are gained at sites characterized by strong GRE motifs, which show GR recruitment in non-liver tissues. The functional importance of these HNF4A-regulated GR sites is indicated by an altered transcriptional response to glucocorticoid treatment in the *Hnf4a*-null liver.

## Highlights

• In mouse liver, nuclear factor HNF4A marks a population of GR binding sites
• Deletion of *Hnf4a* results in loss of GR binding at HNF4A-marked sites
• In the absence of HNF4A, GR binds at response elements not typically bound in liver
• Chromatin accessibility is concurrently remodeled at HNF4A-dependent GR sites

## Graphical abstract

**Figure:**
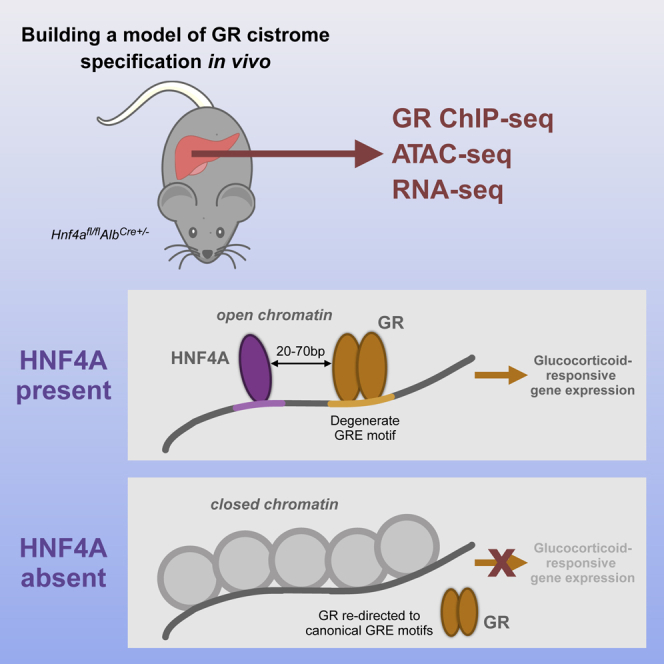

## Teaser

The action of the glucocorticoid receptor (GR) is dependent upon cell type and cell state. Hunter et al. show that, in mouse liver, the lineage-determining factor HNF4A is important for specifying the genome-wide binding profile of GR. Deletion of *Hnf4a* remodels chromatin accessibility and GR binding at HNF4A-marked sites.

## Introduction

NR3C1, the glucocorticoid receptor (GR), is an almost ubiquitously expressed nuclear receptor. While there is evidence for rapid, non-genomic actions of glucocorticoids (Dallman, 2005; Kershaw et al., 2020), the chief outcomes of GR activation occur through its nuclear activity. GR binds the genome through the glucocorticoid response element (GRE) motif, which comprises two palindromic hexamers separated by a 3 bp spacer (AGAACANNNTGTTCT). Formation of higher-order GR structures (dimerization, tetramerization) is necessary for GR-mediated gene regulation (Johnson et al., 2021).

Upon ligand binding, GR is predominantly directed to sites on the genome where chromatin is already accessible (John et al., 2011), and this is dependent on cell type, with priming by C/EBPB (CCAAT-enhancer binding protein beta) particularly important in the liver (Grøntved et al., 2013). GR can also demonstrate pioneer function at sites of inaccessible chromatin (Johnson et al., 2018). While it is less clear what the determinants of binding are here, similarity of the GR-bound DNA sequence to the canonical GRE (“motif strength”) may play a role, with nucleosome-deplete sites demonstrating more degenerate GRE motifs (Johnson et al., 2018). Other studies have shown that active histone marks and presence of pioneer factors also play a role in dictating GR binding (McDowell et al., 2018). Following GR binding, gene activation—involving recruitment of coactivators and chromatin remodelers—occurs at sites of pre-established enhancer-promoter interactions, with the presence of GR increasing the frequency of productive interactions (D’Ippolito et al., 2018). In contrast, the mechanism by which GR downregulates gene expression remains an area of considerable debate, with evidence for protein-protein tethering, indirect mechanisms of action, and GR binding to negative or cryptic response elements presented (Escoter-Torres et al., 2020; Hudson et al., 2018; Miranda et al., 2013; Oh et al., 2017; Sacta et al., 2018; Surjit et al., 2011).

Surprisingly for a transcription factor which is so widely expressed, GR action is remarkably context specific. GR activity can be influenced both by metabolic and immune state (Goldstein et al., 2017; Quagliarini et al., 2019; Uhlenhaut et al., 2013). GR action is also highly tissue specific, with the GR cistrome showing limited overlap between different cell types (John et al., 2011). This is a property which is far from unique to GR, and has been well illustrated for other transcription factors from multiple classes, including the estrogen receptor (Gertz et al., 2013) and the core clock protein BMAL1 (Beytebiere et al., 2019). *In vitro* studies suggest that GR cell specificity is conferred by differential chromatin accessibility at distal enhancer sites, with GR binding in proximal promoter regions regulating genes that are ubiquitously glucocorticoid responsive (Love et al., 2017). In this *in vivo* study, we show the dominance of hepatocyte nuclear factor 4A (HNF4A), itself a nuclear receptor, in determining GR binding in mouse liver. We find the HNF4A motif to underlie sites of GR binding and, in *Hnf4a*-null liver, demonstrate loss of chromatin accessibility and GR binding at HNF4A-marked sites. We see emergence of non-liver-specific GR binding events at sites characterized by strong GRE motifs. We find evidence that this remodeling of the GR cistrome is of functional importance in shaping an altered transcriptomic response to glucocorticoids in the absence of HNF4A.

## Results

### HNF4A motifs mark liver GR binding sites

**Figure 1:**
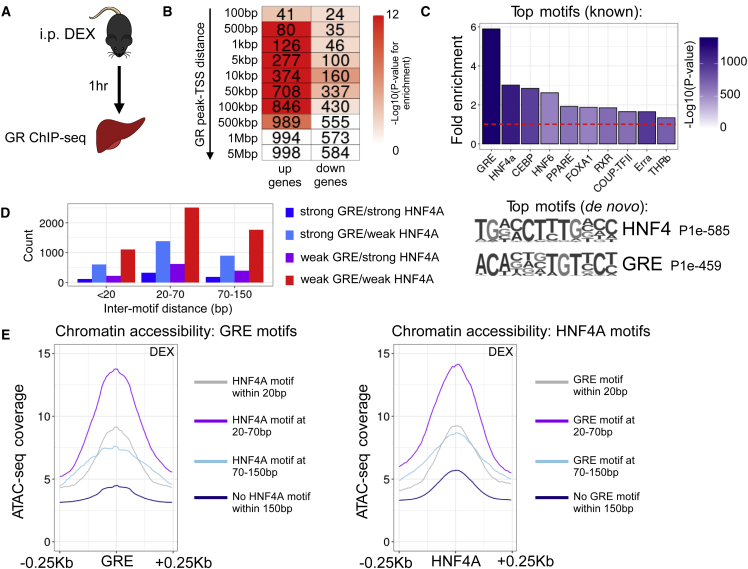
Liver GR binding sites are marked by GRE and HNF4A motifs (A) Liver GR ChIP-seq was performed 1 h after acute dexamethasone (DEX) treatment (n = 2 biological replicates). (B) Heatmap showing enrichment (hypergeometric test) of the transcription start sites (TSSs) of genes up or downregulated by glucocorticoid treatment at increasing distances from GR ChIP-seq peaks. Shading of each cell indicates –log10(p value) for enrichment (over all genes in the genome), number indicates number of genes in each cluster at that distance. (C) Fold enrichment, in GR ChIP-seq peaks, of known motifs. Red dotted line at y = 1. Shading indicates -log10(p value for enrichment). Top motifs found *de novo* shown below. P values for enrichment shown. (D) Bar chart of inter-motif distances for GRE and HNF4A motifs detected within GR ChIP-seq peaks. (E) ATAC-seq coverage score (mean coverage from three biological replicates), in DEX-treated liver, around canonical GRE motifs with or without a HNF4A motif within specified distances (left panel), and around HNF4A motifs with or without a GRE motif within specified distances (right panel). See also Figure S1 and Table S1.

We first mapped the hepatic GR cistrome by performing GR ChIP-seq on mouse liver collected 1 h after intraperitoneal injection of dexamethasone (DEX) at Zeitgeber time 6 (mice housed in 12 h:12 h light-dark cycles, ZT0 = lights on, n = 2) (Figure 1A). As expected, DEX treatment resulted in substantial GR recruitment to the genome, with 20,064 peaks called over input (q < 0.01) in DEX-treated tissue.

We have previously shown that the same model of glucocorticoid treatment has a large effect on the liver transcriptome, with the expression of 1,709 genes being significantly altered (1,052 upregulated, 657 downregulated) (Table S1) (Caratti et al., 2018). We now observed pronounced enrichment of glucocorticoid-upregulated genes in relation to GR binding sites (Figure 1B; hypergeometric test) (Briggs et al., 2021; Yang et al., 2019). Enrichment was seen at distances of 500 bp—500 kbp between transcription start sites of DEX-activated genes and GR ChIP-seq peaks (Figure 1B), implying both proximal and distal regulation. The noticeably weaker enrichment of glucocorticoid-downregulated genes supports the notion, reported by others (Jubb et al., 2016; Oh et al., 2017), that gene downregulation occurs by indirect means; however, we cannot exclude mechanisms, such as tethering, based on these data.

We proceeded to motif discovery analysis, and found the canonical GRE to be the most highly enriched known motif within GR binding sites, followed by the HNF4A motif (Figure 1C; see also Mendeley data: https://doi.org/10.17632/k8d386ndz6.3). A motif most closely resembling the HNF4 motif had the lowest p value on *de novo* motif analysis (Figure 1C), and was detected in 25.64% of GR peaks. As reported for other GR ChIP-seq studies (Hemmer et al., 2019; Lim et al., 2015), other motifs detected (at lower significance) included CEBP, PPAR, and HNF6 motifs. A similar pattern of motif enrichment was seen in GR ChIP-seq peaks called in vehicle-treated liver (n = 2) (5,831 peaks called over input), with the known GRE and HNF4A motifs being the most strongly enriched, and a HNF4-like motif detected *de novo* in 29.29% of peaks (Figures S1A and S1B). Interestingly, we still observed strong enrichment of DEX-upregulated genes at distances of 5–500 kbp from GR peaks in VEH liver (Figure S1C), implying some pre-existing GR binding in association with glucocorticoid-responsive genes, likely reflecting the action of the endogenous GR ligand corticosterone.

We were interested to know how closely GRE and HNF4A motifs were situated at GR binding sites, as the distance between transcription factor (TF) motifs contributes to the likelihood of TFs co-occupying regulatory elements (Sönmezer et al., 2021). Under GR peaks where we observed co-occurrence of HNF4A and GRE motifs, we saw a spread of inter-motif distances (Figure S1D), with 30–50 bp being most frequent, irrespective of whether motif calling was performed with high stringency settings (“strong” GRE/HNF4A), or permitted some degeneracy (“weak”). DNA footprinting approaches (Sönmezer et al., 2021) suggest that TF co-occupancy is most likely at inter-motif distances <20 bp, with a random distribution of co-occupancy events observed at inter-motif distances >70 bp. High levels of co-occupancy could suggest that binding is physically co-operative (Sönmezer et al., 2021). When binned in this way, our data show that inter-motif GRE-HNF4A distances of 20–70 bp are most common (Figure 1D), favoring co-occupancy of GR and HNF4A at regulatory elements, but not necessarily supporting physical co-operation between the two nuclear receptors.

To explore the importance of HNF4A via an independent approach, we performed ATAC-seq (assay for transposase-accessible chromatin) on DEX- and VEH-treated mouse liver. We used HOMER to map the positions of all canonical GREs in the mouse genome (>137,000 locations). At GREs with a nearby HNF4A motif (within 20 bp, 3,281 sites; at 20–70 bp, 2,986 sites; at 70–150 bp, 4,872 sites), we observed stronger mean ATAC signal in DEX-treated liver than at GREs without a nearby HNF4A motif (>125,000 sites) (Figure 1E), with signal strongest at 20–70 bp inter-motif distances. ATAC signals centered around HNF4A motifs (total >567,000 locations) echoed these data, being greatest at HNF4A motifs with a GRE motif within 20–70 bp (Figure 1E). In VEH-treated liver, we observed the same patterns, albeit with an overall reduction in ATAC signal associated with GREs (Figure S1E), likely reflecting the reduction of nuclear (and thus genomic) recruitment of GR in the absence of ligand activation. Therefore, in liver, chromatin accessibility at co-located GRE HNF4A motifs is greater than isolated motifs of GRE, or HNF4A alone, suggesting that HNF4A may play a role in determining chromatin accessibility at GR binding sites, and vice versa. Furthermore, integration with gene expression data indicates that co-located GRE and HNF4A motifs mark regulatory elements where GR acts to upregulate gene expression in the *cis* domain. This is in line with existing theories that GR binds the genome at pre-programmed, DNase-sensitive sites (Grøntved et al., 2013; John et al., 2011; Præstholm et al., 2020), with glucocorticoid treatment augmenting GR action at these sites, and increasing the frequency of pre-established enhancer-promoter interactions (D’Ippolito et al., 2018).

### HNF4A loss remodels the GR cistrome

We thus hypothesized that removing HNF4A would impact upon patterns of GR binding. To test this, we performed GR ChIP-seq on livers from 6- to 8-week-old *Hnf4a*^*fl/fl*^*Alb*^*Cre*^ mice (Hayhurst et al., 2001) treated acutely with DEX, again at ZT6. *Hnf4a*^*fl/fl*^*Alb*^*Cre+/−*^ mice are viable, but show hepatomegaly and hepatosteatosis from age 6 weeks, and increased mortality from age 8 weeks (Hayhurst et al., 2001). Nonetheless, they present a useful, widely used model (Fekry et al., 2018; Karagianni et al., 2020; Wu et al., 2020; Xu et al., 2021) to study how HNF4A regulates transcriptional activity *in vivo*, with loss of liver *Hnf4a* gene expression (Figure S2A) and protein evident (Xu et al., 2021). We performed a differential binding analysis (Lun and Smyth, 2015, 2016) to detect sites where GR ChIP-seq signal was statistically different (false discovery rate [FDR] < 0.05) between *Hnf4a*^*fl/fl*^*Alb*^*Cre−/−*^ (liver “wild type” [LWT]) and *Hnf4a*^*fl/fl*^*Alb*^*Cre+/−*^ (liver knockout [LKO]) livers (n = 2/group). We employed an internal spike-in normalization strategy with *D. melanogaster* chromatin (Egan et al., 2016) to control for technical variation, and so increase confidence that results represented true genotype effects.

**Figure 2:**
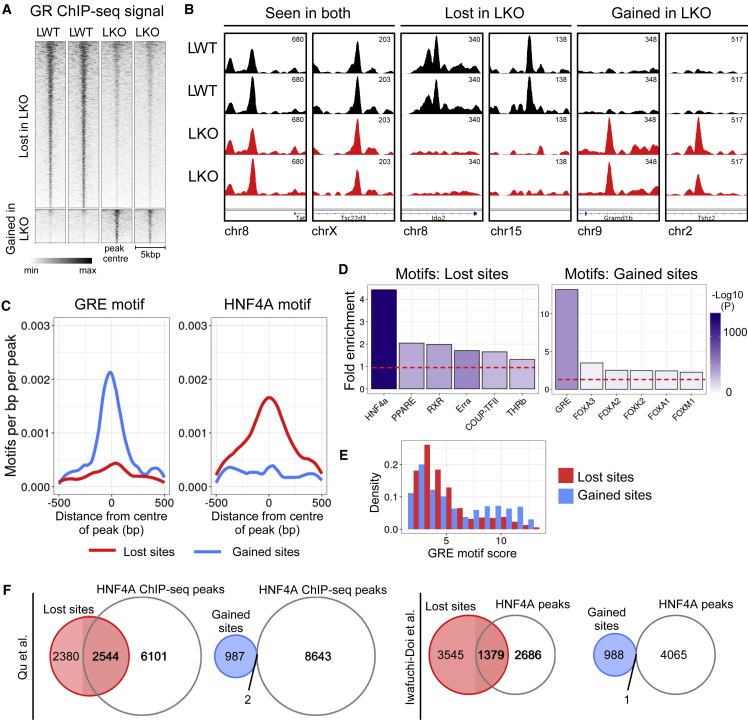
The liver GR cistrome is remodeled in the absence of HNF4A (A) GR ChIP-seq signal at *csaw*-detected DB sites in *Hnf4a*^*fl/fl*^*Alb*^*Cre*^ LWT and LKO mouse liver (signal shown ±2.5 kbp from center of each GR site) (n = 2 biological replicates/genotype). (B) GR ChIP-seq signal tracks of exemplar DB sites. The y axis is uniform within each panel. (C) Abundance of the GRE and HNF4A motifs within lost (red) and gained (blue) GR sites. (D) Fold enrichment, in lost and gained GR sites, of the six most highly enriched known motifs. Red dotted line at y = 1. Shading indicates -log10(p value for enrichment). (E) Density histogram of GRE motif scores (measure of motif strength) in lost (red) and gained (blue) GR sites. (F) Venn diagrams showing overlap of lost and gained GR sites with published HNF4A cistromes from Qu et al. (2018) (left), and Iwafuchi-Doi et al. (2016) (right). See also Figure S2.

This approach detected 4,924 sites where GR binding was lost in LKO animals compared with LWT (FDR < 0.05), and 989 sites where GR binding was gained. Loss of HNF4A therefore led to substantial remodeling of the liver GR cistrome (Figures 2A and 2B). Lost and gained sites chiefly annotated to intergenic and intronic regions of the genome, suggesting that remodeling was affecting distal regulatory sites rather than proximal promoter regions (Figure S2B). In keeping with previous work (Love et al., 2017), this supports the notion that tissue specificity of GR action may be largely conferred by distal enhancers, rather than proximal promoter binding.

To examine the distinctions between lost and gained GR sites in more detail, we first quantified the abundance of specific motifs. We observed that GR sites lost in LKO liver showed low abundance of the canonical GRE and high abundance of the HNF4A motif, while GR sites gained showed high abundance of the canonical GRE and low HNF4A motif abundance (Figure 2C). These findings were recapitulated by motif discovery analysis (Figure 2D; see also Mendeley data: https://doi.org/10.17632/k8d386ndz6.3); the HNF4A motif being the most strongly enriched in lost GR sites supports the specificity of the effect. On comparing the strength of GRE motifs in lost and gained sites (scored by similarity to the canonical GRE), we found lost GR sites to be predominantly characterized by weak GREs, while strong GREs characterized a population of gained sites (Figure 2E). Within lost sites, co-occurrence of GRE and HNF4A motifs was most numerous at inter-motif distances of 20–70 bp (Figure S2C), as we observed for the wider GR cistrome. Comparison of our data with recently published HNF4A liver cistromes demonstrated overlap of lost GR sites with HNF4A binding sites (Figure 2F), suggesting that the HNF4A protein, in addition to the motif, can normally be found at these sites. Unsurprisingly, we saw almost no overlap of gained GR sites with the HNF4A cistrome.

Therefore, in the absence of HNF4A, GR is no longer recruited to sites marked by the HNF4A motif (and HNF4A binding) and a weak GRE motif. Intriguingly, GR binding emerges at sites where it is not normally recruited, where strong GRE motifs are present (and where HNF4A is not found). This marked divergence between lost and gained sites points to this being a specific consequence of HNF4A loss, and not a downstream effect of the abnormal hepatic physiology of *Hnf4a*^*fl/fl*^*Alb*^*Cre+/−*^ livers.

### HNF4A-dependent GR sites demonstrate distinct patterns of chromatin accessibility and tissue GR recruitment

**Figure 3:**
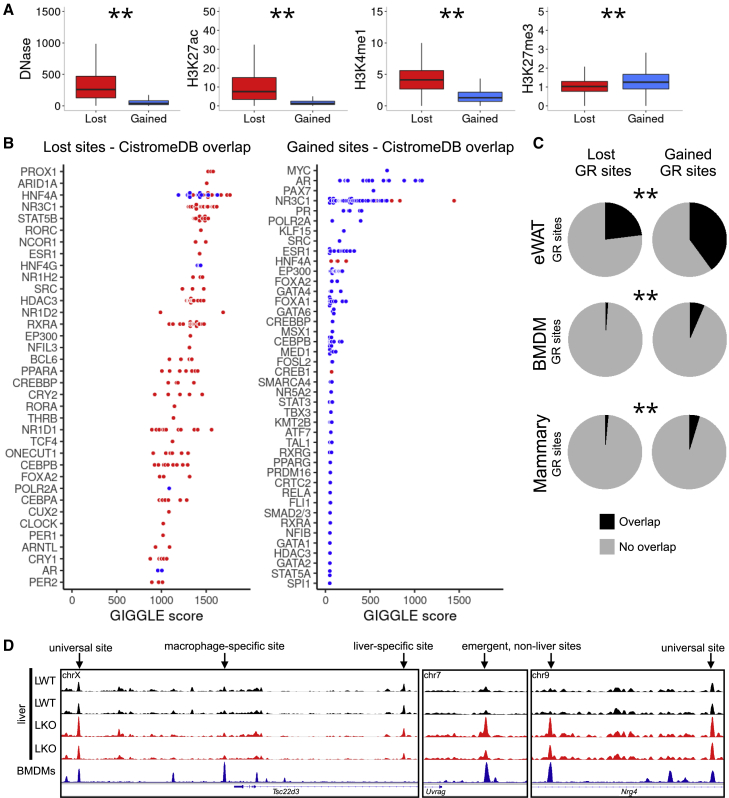
Lost and gained GR sites diverge by chromatin state and tissue specificity (A) Box-and-whisker plots showing read coverage of lost and gained GR sites of signal from DNase-seq and ChIP-seq of histone marks H3K27ac, H3K4me1, H3K27me3. ^∗∗^p < 0.01, Wilcoxon tests. Central line at median, box limits at 25th and 75th percentiles, whiskers extend 1.5× interquartile range from box limits. (B) Overlap of lost and gained GR sites with published transcription factor cistrome data (top 1k peaks in each dataset), as determined and scored by GIGGLE. Datasets from non-liver tissues/cells plotted in blue, datasets from liver/hepatocytes plotted in red. (C) Overlap of lost and gained sites with GR binding sites in epididymal white adipose tissue (eWAT) (Soccio et al., 2015), bone marrow-derived macrophages (BMDMs) (Oh et al., 2017), and mammary tissue (Shin et al., 2016). ^∗∗^p < 0.01, chi-square tests. (D) Exemplar tracks showing GR ChIP-seq signal around the *Tsc22d*3 (*Gilz*), *Uvrag*, and *Nrg4* loci in LWT and LKO liver, and in bone marrow-derived macrophages (Oh et al., 2017). Universal, macrophage-specific and liver-specific GR sites highlighted by arrows. The y axis is uniform within each panel.

Next, we sought to understand the normal chromatin state of GR sites lost or gained in the absence of HNF4A. We took advantage of published datasets for DNase hypersensitivity (Sobel et al., 2017) and histone mark ChIP-seq (Armour et al., 2017; Shen et al., 2012) in naive mouse liver (i.e., the chromatin landscape that the GR encounters upon dexamethasone treatment) to quantify read coverage at lost and gained sites. We found that GR is lost at sites where, in liver, chromatin is normally DNase sensitive, and where higher levels of the histone marks H3K27ac and H3K4me1—associated with active/poised enhancers—are found. GR binding is gained at sites where chromatin is normally less DNase sensitive, and where the H3K27me3 mark (associated with inactive regions of heterochromatin) is stronger (Figure 3A).

Given that HNF4A is a lineage-determining TF, we hypothesized that loss of HNF4A results in a GR cistrome less specific to the liver. We performed an unbiased comparison of lost and gained sites with TF cistromes, using the GIGGLE tool (Layer et al., 2018). We found lost sites to be normally bound by not only HNF4A itself, but by multiple other factors with important metabolic roles, with almost all of these cistromes being derived from liver/hepatocyte experiments (Figure 3B; see also Mendeley data: https://doi.org/10.17632/k8d386ndz6.3). In marked contrast, gained sites were most numerously bound by GR (NR3C1) and other NR3 family members, but almost exclusively in non-liver tissues. Importantly, these tissues were diverse, and not simply non-hepatocyte cell types (e.g., inflammatory cells) that might be found within liver tissue. Interestingly, those liver GR cistromes that did show overlap with gained sites (Figure 3B) were from a study where overexpression of a dominant negative form of C/EBP was used to disrupt the liver GR cistrome (Grøntved et al., 2013).

Thus, these findings suggest that HNF4A is necessary for directing GR binding at a population of liver-specific sites by means of maintaining open chromatin. The GREs at these HNF4A-dependent sites show degeneracy from the canonical AGAACANNNTGTTCT motif, but chromatin state is favorable toward GR binding, being marked by active histone modifications. It may be that other important regulators of liver energy metabolism are also recruited to these regions. By contrast, in the absence of HNF4A, GR is recruited to additional sites where chromatin is not normally accessible in liver, but where a strong GRE motif is found, and where GR is capable of binding in other tissues. Indeed, we observed significantly greater overlap between gained GR sites and GR cistromes in non-liver tissues (white adipose, macrophages, mammary gland), than between lost GR sites and non-liver GR cistromes (Figures 3C and 3D).

### In the absence of HNF4A, chromatin accessibility is remodeled at HNF4A-dependent GR sites

**Figure 4:**
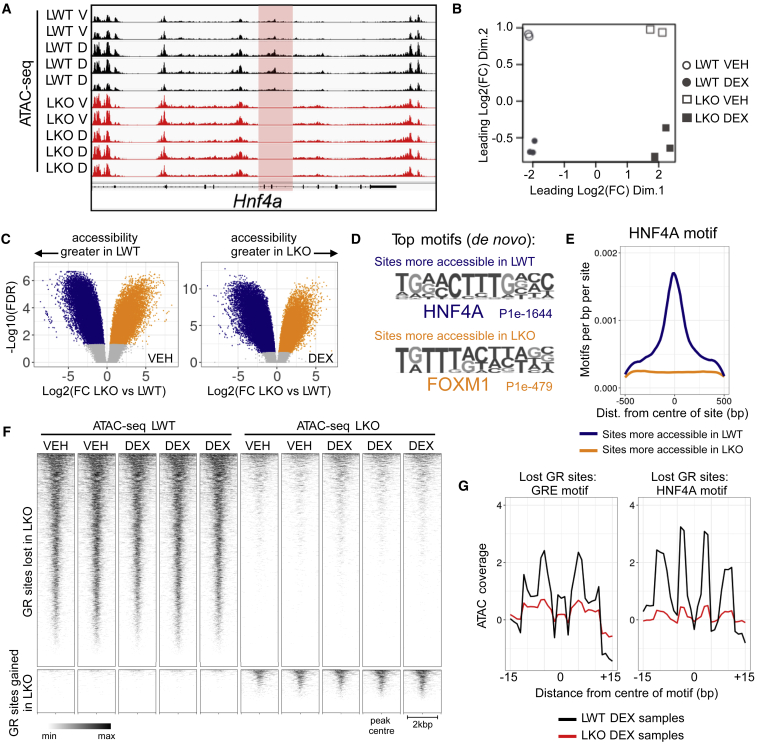
Altered chromatin accessibility in *Hnf4a*-null liver associates with the remodeled GR cistrome (A) ATAC-seq signal in all 10 samples sequenced, showing excision of exons 4 and 5 of the *Hnf4a* gene in *Hnf4a*^*fl/fl*^*Alb*^*Cre*^ LKO mice. The y axes are uniform. (B) Multidimensional scaling plot of liver ATAC-seq samples demonstrates a large effect of genotype (dimension 1), and an effect of dexamethasone treatment (dimension 2). Each point represents one biological replicate (n = 2–3/group). (C) Differential accessibility analysis with *csaw* finds >30,000 sites where chromatin accessibility is significantly remodeled (FDR < 0.05) by *Hnf4a* loss in vehicle-treated mice, >34,000 sites in dexamethasone-treated mice. Sites with significantly increased accessibility in LWT mice highlighted in navy, sites with significantly increased accessibility in LKO mice in orange. (D) *De novo* motif analysis finds an HNF4A-like motif detected most strongly (lowest p value) at sites where chromatin accessibility is greater in intact mice (LWT), while a FOXM1-like motif is detected at sites where chromatin is more accessible in *Hnf4a*-null mice (LKO). P values for enrichment shown. (E) Abundance of the HNF4A motif at LWT sites (navy), and at LKO sites (orange). (F). ATAC-seq signal at sites of altered GR binding in *Hnf4a*^*fl/fl*^*Alb*^*Cre*^ LWT and LKO mouse liver (signal shown ± 1 kbp from center of each GR site). (G) ATAC-seq signal coverage (to 1 bp resolution) in LWT (black) and LKO (red) liver, at GRE and HNF4A motifs within sites of lost GR binding.

To test the hypothesis that GR binding is determined by HNF4A-dependent chromatin accessibility, we proceeded to ATAC-seq to map transposase-accessible sites in intact and *Hnf4a*-null liver (VEH and DEX treated), across a total of ten samples. As expected, ATAC-seq confirmed excision of *Hnf4a* exons 4 and 5 in *Hnf4a*^*fl/fl*^*Alb*^*Cre*^ LKO mice (Figure 4A). On performing differential accessibility analysis with *csaw*, we saw a stark distinction between LWT and LKO samples (Figure 4B), indicating that HNF4A loss leads to substantial remodeling of the chromatin environment. In LKO mouse liver, compared with LWT, chromatin accessibility was significantly reduced (FDR < 0.05) at 16,103 sites in VEH-treated animals, and significantly increased at 14,531 sites (Figure 4C). In DEX-treated LKO animals, 17,400 sites showed reduced accessibility and 16,691 sites increased accessibility. We also saw that glucocorticoid treatment affected chromatin accessibility (Figure 4B), with DEX treatment increasing accessibility at GRE- and CEBP-marked sites (Mendeley data: https://doi.org/10.17632/k8d386ndz6.3).

We proceeded to more detailed motif analysis of sites where chromatin accessibility was impacted by *Hnf4a* deletion. Motif discovery found sites with greater accessibility in LWT mice (i.e., reduced in LKO) to be strongly enriched for the HNF4A motif (Figures 4D and 4E; see also Mendeley data: https://doi.org/10.17632/k8d386ndz6.3), while sites with greater accessibility in LKO mice were enriched for Forkhead box (FOX) motifs (FOXM1; Figure 4D; see also Mendeley data: https://doi.org/10.17632/k8d386ndz6.3). These data indicate that loss of HNF4A results in reduced chromatin accessibility primarily at HNF4A binding sites, and increased chromatin accessibility at sites marked by FOX factors.

We thus hypothesized that the remodeling of the GR cistrome seen with *Hnf4a* deletion chiefly reflects remodeling of the chromatin environment. Importantly, the chromatin remodeling we observed was of a different order of magnitude to the remodeling of the GR cistrome (>30,000 loci with altered ATAC-seq signal, compared with ∼5,000 loci with altered GR binding). This implies that the impact of HNF4A loss extends beyond the GR cistrome and, indeed, this is corroborated by recent work showing large changes in the BMAL1 cistrome with *Hnf4a* deletion (Qu et al., 2021).

On quantifying ATAC signal at sites of differential GR binding, chromatin accessibility positively correlated with GR binding (Figure 4F); where GR binding is lost in LKO animals, we observed reduction of chromatin accessibility; where GR binding is gained, we saw increased chromatin accessibility. A total of 77% of lost GR sites (3,777 sites) directly overlapped with the 17,400 sites of significantly decreased chromatin accessibility; 57% of gained GR sites (568 sites) overlapped with the 16,691 sites of increased accessibility (the incomplete overlap of altered GR sites with altered accessibility sites likely results from false negatives and false positives in each group of sites). Supporting concurrent loss of HNF4A binding at the lost GR sites, we saw a marked alteration in the ATAC-seq signal “footprint” from LKO samples around HNF4A motifs (Figure 4G).

Our GR ChIP-seq and ATAC-seq data together indicate, therefore, that HNF4A loss in *Hnf4a*^*fl/fl*^*Alb*^*Cre*^ LKO liver reduces accessibility of chromatin at sites usually marked by HNF4A, reducing GR recruitment to co-located weak GREs. Increased accessibility at FOX-marked sites, associated with strong GREs, increases GR recruitment to these loci.

### HNF4A loss remodels the glucocorticoid-responsive transcriptome

**Figure 5:**
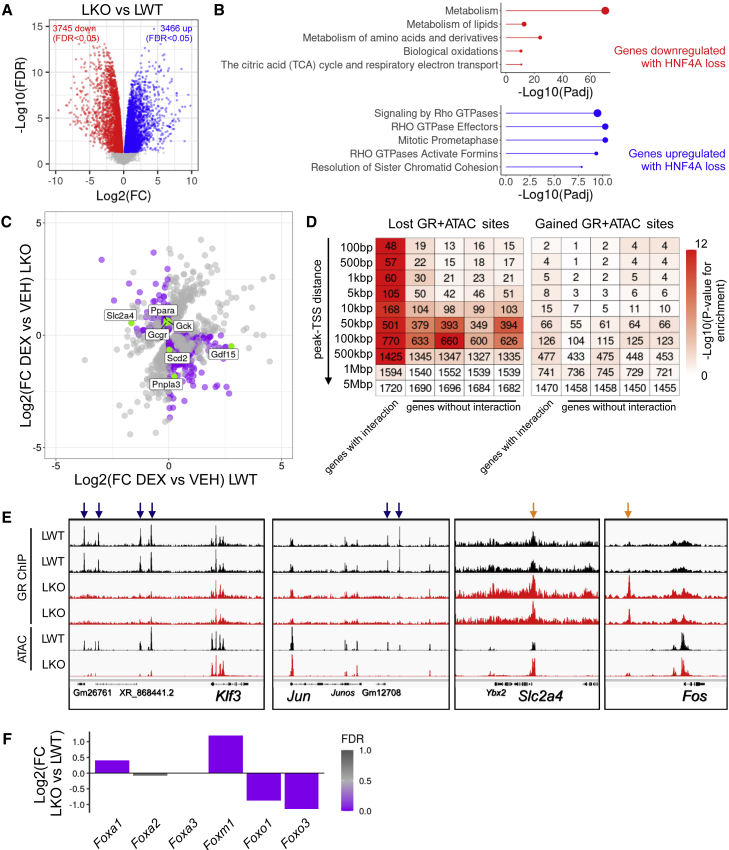
Remodeled GR sites associate with genes showing altered glucocorticoid response (A) Liver RNA-seq in vehicle-treated *Hnf4a*^*fl/fl*^*Alb*^*Cre*^, LKO versus LWT samples. Significantly downregulated genes (FDR < 0.05) in red, significantly upregulated genes in blue (n = 3–4 biological replicates/group). (B) Top Reactome pathways of genes downregulated (red) and upregulated (blue) by HNF4A loss. Point size is proportional to number of genes in that pathway. P value from hypergeometric test, Benjamini Hochberg adjusted. (C) Effect of DEX treatment in LWT and LKO mice (n = 3–4 biological replicates/group). Genes where stageR detects a significant treatment × genotype interaction shown in gray (1,908 genes). Those where direction of change is different between genotypes, and where effect of treatment is significant, highlighted in purple (633 genes). These include notable metabolic regulators and enzymes, highlighted in green. FC, fold change. (D) Enrichment of gene clusters at increasing distances from sites where GR binding and chromatin accessibility is lost or gained in *Hnf4a*^*fl/fl*^*Alb*^*Cre*^ LKO mice (hypergeometric test). First cluster comprises those 1,908 genes where stageR detects a treatment-genotype interaction, other clusters comprise random samples of equivalent size (repeated four times) of DEX-responsive genes where no treatment-genotype interaction is detected. Shading of each heatmap cell corresponds to –log10(p value) for enrichment, number indicates number of genes in each cluster at that distance. (E) Exemplar tracks showing GR ChIP-seq and ATAC-seq signals around the *Klf3*, *Jun*, *Slc2a4*, and *Fos* loci in LWT and LKO liver. Sites of lost GR binding and chromatin accessibility indicated with navy arrows, sites of gained GR binding and chromatin accessibility indicated with orange arrows. The y axis is uniform within each panel. (F) Hepatic expression of Forkhead box factor genes in *Hnf4a*^*fl/fl*^*Alb*^*Cre*^ LKO compared with LWT mice. FDR, false discovery rate. See also Tables S2 and S3.

To examine the functional importance of this GR cistrome remodeling, we then performed liver RNA-seq to quantify gene expression in dexamethasone- and vehicle-treated *Hnf4a*^*fl/fl*^*Alb*^*Cre*^ LWT and LKO mice (n = 3–4/group). Simply by studying differential gene expression in vehicle-treated mice, we saw a profound effect of HNF4A on the liver transcriptome (Figure 5A), with the expression of >7,000 genes being different between genotypes. Genes with diminished expression in LKO mice were characterized by pathways of lipid, amino acid, and oxidative metabolism, while genes with increased expression in LKO mice were associated with non-liver pathways, such as Rho GTPase (intracellular actin dynamics) and cell-cycle pathways (Figure 5B). This loss of liver identity with *Hnf4a* deletion echoes observations in models of non-alcoholic steatohepatitis where, interestingly, ATAC-seq analyses indicate loss of HNF4A activity (Loft et al., 2021).

Importantly for our hypothesis, loss of HNF4A also altered the response to acute glucocorticoid treatment. By comparing gene expression in LKO and LWT mice treated with dexamethasone or vehicle, using R Bioconductor package stageR (Van den Berge et al., 2017), specifically designed to control FDR at the gene level, we found 1,908 genes where a significant genotype-treatment interaction was detected (adjusted p value < 0.05). Of these 1,908 genes, 633 showed a marked difference in response to DEX treatment between the two genotypes (Figure 5C). Of note, these included important metabolic genes (e.g., *Ppara*, *Gdf15*, *Gck*), suggesting that HNF4A exerts a major impact on the liver metabolic response to glucocorticoids. *Gdf15* expression, for example, is normally upregulated by glucocorticoid, an effect that is lost in the *Hnf4a*^*fl/fl*^*Alb*^*Cre+/−*^ mice. Pathway analysis of DEX-upregulated genes with a treatment-genotype interaction (Tables S2 and S3) detected enrichment of cholesterol biosynthesis and steroid metabolism processes, suggesting that glucocorticoid regulation of these pathways might be under HNF4A control, specifically targeting these actions to the liver.

To determine whether these changes in transcriptomic response might directly relate to the remodeling of the GR cistrome, we looked for significant enrichment of genes of interest in the locale of lost and gained GR sites also marked by significant change in chromatin accessibility (3,777 lost sites, 568 gained sites). Specifically, we looked at the 1,908 genes where stageR detected a significant treatment-genotype interaction, and asked whether these were over-represented in proximity to these GR + ATAC sites (Figure 5D). As control clusters, we took DEX-responsive genes where stageR did not detect such an interaction (3,797 genes) and randomly sampled 1,908 genes from this group, repeating this random sampling four times.

At GR + ATAC sites lost in the absence of HNF4A, at distances between 100 bp and 500 kbp, we saw strong enrichment of genes with a treatment-genotype interaction (e.g., *Klf3* and *Jun*, Figures 5D and 5E). Interestingly, strong of genes without an interaction was also apparent, but at distances of 50–100 kbp from lost GR sites (Figure 5D). It may be that a proportion of these sites are distal enhancers that make a redundant contribution to the regulation of the genes in question, or a contribution that is sufficiently small not to be detected in our RNA-seq analysis. Other, HNF4A-independent regulatory elements may exert more dominant control over these genes.

For GR + ATAC binding sites gained in LKO liver, we observed some genes with a treatment-genotype interaction (e.g., *Slc2a4*, *Fos*) to lie in proximity to gained sites (Figure 5E), but did not observe strong enrichment at the genome-wide level (Figure 5D), which may be a reflection of the small number of sites involved. Our results support the idea that loss of GR binding in *Hnf4a*-null liver contributes to the observed alteration in the transcriptional response to glucocorticoid treatment, and that the remodeling of the GR cistrome is of functional importance. While we are not able to definitively prove a cause-effect relationship, we see strong statistical associations across thousands of sites and thousands of genes. Gained GR sites may also alter the glucocorticoid response, but with an effect size that is masked by the large impact of *Hnf4a* deletion on the global transcriptome.

Given the emergence of GR binding and chromatin accessibility at sites marked by FOX TFs (Figures 2D and 4D), we specifically looked at expression of FOX factor genes (Figure 5F). FOX proteins have been proposed to have pioneer function, and co-localize with steroid hormone receptors (Sanders et al., 2013; Zhang et al., 2005). Of note, we saw increased expression of *Foxm1* (*Hnf3*) in LKO liver, compared with LWT. Altered FOXM1 activity may therefore contribute to the chromatin remodeling observed with HNF4A loss.

### HNF6 has limited influence on liver GR action

Finally, we were also interested to examine the influence of a hepatic lineage-determining factor from another family. We have found HNF4A deletion to have a substantial effect on GR binding, and others have demonstrated the importance of the bZIP TF C/EBPB (Grøntved et al., 2013). HNF6 emerged in our early analysis of motifs related to GR binding sites (Figure 1C). HNF6 (*Hnf6*), is a lineage-determining factor that is part of the onecut family of TFs (Odom et al., 2004). Although its motif was enriched at hepatic GR binding sites (Figure 1C), it was found at a smaller proportion of GR sites than the HNF4A motif, and its presence in the vicinity of GREs is not associated with the large increase in chromatin accessibility seen with the HNF4A motif (Figure S3A).

We therefore hypothesized that HNF6 plays a more minor role in shaping liver GR action, although HNF6 is required for embryonic development and liver specification. We used a mouse model of postnatal liver *Hnf6* deletion (its embryonic loss is lethal) (Figure S3B). We found that this had only a small effect on the liver transcriptome under basal conditions (Figure S3C), with a correspondingly minor impact on glucocorticoid responsiveness. Analysis with stageR detected 148 genes with a significant treatment-genotype interaction, of which 34 showed an altered direction of significant change with glucocorticoid treatment (Figure S3D). These data suggest that HNF6 is indeed less influential than HNF4A in shaping the response to glucocorticoid, with a lesser functional impact evident. By contrast, our data do support a role for HNF4A in shaping the tissue specificity of GR action. We suggest that, as a lineage-determining factor, HNF4A confers tissue specificity to the liver GR cistrome by maintaining chromatin accessibility at HNF4A motif-marked sites (assisted loading). In the absence of HNF4A, the regulatory landscape is remodeled, and GR binds to strong canonical GRE motifs normally inaccessible in the terminally differentiated hepatocyte.

## Discussion

In this study, we show that a substantial portion of the liver GR cistrome is characterized by HNF4A binding and the HNF4A motif. The presence of the HNF4A motif favors open chromatin, in comparison to sites where the HNF4A motif is not present. Strikingly, when HNF4A is removed, the chromatin environment and thus the GR cistrome are remodeled, with the HNF4A motif enriched at sites where GR binding is lost. Furthermore, GR binding emerges at sites where GR is typically bound in non-liver tissues where chromatin is not normally accessible in liver. At these sites, we see enrichment of FOX factor motifs, notably FOXM1, whose gene expression is also upregulated in *Hnf4a*-null liver.

Multiple previous studies have demonstrated the presence of the HNF4A motif at GR binding sites (Caratti et al., 2018; Hemmer et al., 2019; Lim et al., 2015; Quagliarini et al., 2019), and have shown the tissue specificity of nuclear receptor cistromes (Gertz et al., 2013; John et al., 2011). C/EBPB and the basic-helix-loop-helix factor E47 have also been shown to play important roles in regulating hepatic glucocorticoid action (Grøntved et al., 2013; Hemmer et al., 2019). This study builds on these works by directly comparing the GR cistrome in *Hnf4a*-intact and *Hnf4a*-null liver, identifying those GR binding sites that are dependent on HNF4A, and showing that loss of tissue specificity extends to the emergence of “non-liver” GR binding sites. Furthermore, we show that sites marked by the HNF4A motif, and those sites lost and gained in *Hnf4a*-null liver, have distinct profiles of chromatin accessibility.

The characteristics of the GR sites that are gained and lost in the absence of HNF4A suggest a balance between chromatin accessibility (John et al., 2011) and GRE motif strength (Johnson et al., 2018) in specifying GR binding. Numbers of DNA-bound GR molecules per cell are thought to be in the order of the hundreds (Paakinaho et al., 2017), and are thus outnumbered by the number of potential GR binding sites (motif analysis suggesting >137,000 GREs across the mouse genome). Where HNF4A maintains chromatin accessibility, GR may bind to a weak motif with considerable degeneracy from the canonical GRE. When HNF4A is not present, GR no longer binds these sites, but can instead bind strong GRE motifs at sites where chromatin is not normally accessible in liver (Figure S4). GR has been proposed to have intrinsic pioneer function (Johnson et al., 2018), able to induce chromatin opening at inaccessible sites. Interestingly, our data are compatible with recent work on FOXA1 and HNF4A, which suggests that pioneer function is not a binary property of transcription factors but, rather, that TF binding is determined by its intrinsic physical properties and motif availability (Hansen et al., 2022). In a related fashion, major perturbations of the regulatory environment that likely induce chromatin remodeling (e.g., fasting [Goldstein et al., 2017], chronic high fat diet [Quagliarini et al., 2019]) have been shown to alter the observed actions of GR, and we suggest that, operating through a similar mechanism, this phenomenon extends to other nuclear receptors whose activity is state sensitive (Hunter et al., 2020; Zhang et al., 2017). Indeed, the “cistromic plasticity” of the estrogen receptor is proposed to be of clinical importance in breast cancer (Mayayo-Peralta et al., 2021).

While HNF4A and GR have been identified together in ChIP-MS studies (Quagliarini et al., 2019), our data suggest a permissive role for HNF4A akin to what is proposed for C/EBPB—that of maintaining chromatin accessibility at commonly occupied sites—rather than direct co-operative interaction between the two nuclear receptors. When HNF4A is lost, we see greatly reduced chromatin accessibility at HNF4A-marked sites. Recent complementary recent work has also demonstrated loss of active histone marks H3K4me1 and H3K27ac in *Hnf4a*^*fl/fl*^*Alb*^*Cre*^ LKO liver (Qu et al., 2021). In addition, there is a broad distribution of inter-motif distances, with many GRE-HNF4A motif pairs lying further apart than the 20 bp proposed for high-confidence co-occupancy (and thus physical co-operativity) (Sönmezer et al., 2021). There are clearly many sites where GR binding is not dependent on HNF4A, and more dynamic context specificity of GR action will also be conferred by the ultradian and circadian variation in the availability of its endogenous ligand (Conway-Campbell et al., 2012; Ince et al., 2019). Thus, the combinatorial actions of lineage-determining factors, state-sensitive factors or chromatin remodeling enzymes, and the rhythmicity of its ligand, confer exquisite context specificity to GR action, and must be taken into account when considering therapeutic applications.

### Limitations of the study

This study demonstrates, *in vivo*, the remodeling of the GR cistrome with the deletion of a lineage-determining factor. Our data echo the results of previous tissue-tissue comparisons of nuclear receptor binding (Gertz et al., 2013), but now show directly the importance of a single factor. This value of the study is inextricably linked to a confounding factor, that of the abnormal liver function that results from disruption of HNF4A expression. The livers of *Hnf4a*^*fl/fl*^*Alb*^*Cre*^ LKO mice are enlarged, demonstrating hepatocyte hypertrophy and lipid accumulation (Hayhurst et al., 2001). This makes it difficult to perform more detailed physiological studies in this line. However, we mitigated the liver pathology as much as possible by studying animals at a young age, in what is a widely used mouse line (Fekry et al., 2018; Karagianni et al., 2020; Wu et al., 2020; Xu et al., 2021). Sites where GR binding is lost enrich most strongly for the HNF4A motif (this is also the case for sites of reduced chromatin accessibility), and overlap with HNF4A binding sites, supporting an effect specific to HNF4A loss, rather than attendant liver pathology. Undoubtedly, however, some remodeling of the GR cistrome will reflect indirect rather than direct effects of *Hnf4a* deletion (e.g., those sites that do not overlap with the HNF4A cistrome; Figure 2F). This is a caveat of using this *in vivo* model. Unfortunately, alternative approaches using primary cell culture are difficult, due to rapid loss of differentiated cell function, and liver cancer cell lines offer poor models of liver biology. Similarly, the large impact of *Hnf4a* deletion on the liver transcriptome makes it challenging to distinguish which gene expression changes are specifically due to the loss of HNF4A regulation of GR action. We have taken a statistical association approach (Briggs et al., 2021) to link remodeled GR sites to genes with altered DEX response, which does not prove cause-effect, but does have the advantage of incorporating thousands of genomic loci, in an unbiased manner. Cause and effect might be inferred by mutating or deleting putative enhancers in a hepatic cell line. However, testing the number of remodeled GR sites we detect would require a massively parallel reporter assay (testing a smaller number of sites might introduce selection bias), and we cannot be certain that the chromatin environment in an artificial system would be representative of that *in vivo*. Thus we limit our conclusions to the likelihood that the remodeling of the GR cistrome is of functional importance.

## STAR★Methods

### Key resources table

**Table:** 

REAGENT or RESOURCE	SOURCE	IDENTIFIER
**Antibodies**		

Rabbit polyclonal anti-glucocorticoid receptor	ProteinTech	Cat#24050-1-AP, lot 00044414; RRID: AB_2813890
Rabbit monoclonal anti-glucocorticoid receptor	Cell Signaling	Cat#D8H2, lot 2; RRID: AB_11179215
Spike-In Antibody	Active Motif	Cat#61686, lot 34216004; RRID: AB_2737370

**Chemicals, peptides, and recombinant proteins**		

Dexamethasone (water-soluble)	Sigma Aldrich	Cat#D2915
(2-hydroxypropyl)-beta-cyclodextrin	Sigma Aldrich	Cat#H107
Spike-In Chromatin	Active Motif	Cat#53083
Tamoxifen	Sigma Aldrich	Cat#T5648

**Critical commercial assays**		

ChIP-IT High Sensitivity kit	Active Motif	Cat#53040
TruSeq ChIP-seq Library Preparation kit	Illumina	Cat#IP-202-1012
ATAC-seq kit	Active Motif	Cat#53150
Collibri Library Quantification kit	Thermo Fisher Scientific	Cat#A38524100
Reliaprep RNA Miniprep system	Promega	Cat#Z6111
TruSeq Stranded mRNA kit	Illumina	Cat#20020594
High Capacity RNA-to-cDNA kit	Applied Biosystems	Cat#4387406
PowerUp SYBR Green Master Mix	Thermo Fisher Scientific	Cat#A25741

**Deposited data**		

Mouse reference genome mm10	Genome Reference Consortium	https://www.ncbi.nlm.nih.gov/assembly/GCF_000001635.20/
Drosophila reference genome dm6	Genome Reference Consortium	https://www.ncbi.nlm.nih.gov/assembly/GCF_000001215.4/
Mouse liver DNase-seq (ZT6)	Sobel et al., (2017)	Sequence Read Archive: SRR1551954
Mouse liver H3K27ac ChIP-seq	Armour et al. (2017)	Sequence Read Archive: SRR5054771
Mouse liver H3K4me1 ChIP-seq	Shen et al. (2012)	Sequence Read Archive: SRR317236, SRR317235
Mouse liver H3K27me3 ChIP-seq	Shen et al. (2012)	Sequence Read Archive: SRR566941, SRR566942
Mouse liver HNF4A ChIP-seq	Iwafuchi-Doi et al. (2016)	Sequence Read Archive: SRR7634103, SRR7634104, SRR7634105
Mouse liver HNF4A ChIP-seq	Qu et al. (2018)	Sequence Read Archive: SRR3151870, SRR3151871, SRR3151878, SRR3151879
Mouse macrophage GR ChIP-seq	Oh et al. (2017)	Sequence Read Archive: SRR5182692
Mouse white adipose tissue GR ChIP-seq	Soccio et al. (2015)	Sequence Read Archive: SRR1732507
Mouse mammary gland GR ChIP-seq	Shin et al. (2016)	Sequence Read Archive: SRR3317323
Raw mouse liver GR ChIP-seq data	This paper	ArrayExpress: E-MTAB-10224
Raw mouse liver ATAC-seq data	This paper	ArrayExpress: E-MTAB-10266
Raw mouse liver RNA-seq data	This paper	ArrayExpress: E-MTAB-10247
Analysed ChIP, ATAC, RNA-seq data	This paper	Mendeley Data: (URL: https://data.mendeley.com/datasets/k8d386ndz6/3; DOI: https://doi.org/10.17632/k8d386ndz6.3)

**Experimental models: Organisms/strains**		

Mouse: Hnf4a^fl/fl^Alb^Cre^: B6.129X1(FVB)-Hnf4a^tm1.1Gonz^/Hnf4a^tm1.1Gonz^; Tg(Alb1-cre)1Dlr Mus musculus	Frank J. Gonzalez; Hayhurst et al. (2001).	RRID: MGI:3,653,184
Mouse: Hnf6^fl/fl^: B6-Onecut1^tm1.1Mga/Mmnc^ Mus musculus	MMRRC; Zhang et al. (2009)	RRID: MMRRC_029869-UNC
Mouse: Alb^CreERT2^: B6-Alb^tm1(cre/ERT2)Mtz^ Mus musculus	Daniel Metzger; Schuler et al. (2004)	RRID: MGI:3053224

**Oligonucleotides**		

qPCR primer: *Hnf6* forward: GGCAACGTGAGCGGTAGTTT	PrimerBank	https://pga.mgh.harvard.edu/primerbank/
qPCR primer: *Hnf6* reverse: TTGCTGGGAGTTGTGAATGCT	PrimerBank	https://pga.mgh.harvard.edu/primerbank/
qPCR primer: *Hnf4a* forward: AGAAGATTGCCAACATCAC	PrimerBank	https://pga.mgh.harvard.edu/primerbank/
qPCR primer: *Hnf4a* reverse: GGTCATCCAGAAGGAGTT	PrimerBank	https://pga.mgh.harvard.edu/primerbank/
qPCR primer: *Actb* forward: GGCTGTATTCCCCTCCATCG	PrimerBank	https://pga.mgh.harvard.edu/primerbank/
qPCR primer: *Actb* reverse: CCAGTTGGTAACAATGCCATGT	PrimerBank	https://pga.mgh.harvard.edu/primerbank/

**Software and algorithms**		

FastQC v0.11.7	Babraham Bioinformatics	https://www.bioinformatics.babraham.ac.uk/projects/fastqc/; RRID: SCR_014583
FastQ Screen v0.9.2	Wingett and Andrews (2018)	https://www.bioinformatics.babraham.ac.uk/projects/fastq_screen/; RRID: SCR_000141
Trimmomatic v0.36, v0.38	Bolger et al. (2014)	http://www.usadellab.org/cms/?page%20=%20trimmomatic; RRID: SCR_011848
Bowtie2 v2.3.4.3	Langmead and Salzberg (2012)	http://bowtie-bio.sourceforge.net/bowtie2/; RRID: SCR_016368
STAR v2.5.3a	Dobin et al. (2013)	https://github.com/alexdobin/STAR; RRID: SCR_004463
SAMtools v1.9	Li et al. (2009)	http://www.htslib.org/; RRID: SCR_002105
Picard v2.18.14	Broad Institute	https://broadinstitute.github.io/picard/; RRID: SCR_006525
sratoolkit v2.9.2	NCBI	https://hpc.nih.gov/apps/sratoolkit.html
PEPATAC v0.9.15	Smith et al. (2021)	http://pepatac.databio.org/en/latest/
MACS2 v2.1.1.20160309	Zhang et al. (2008)	https://github.com/taoliu/MACS; RRID: SCR_013291
HOMER v4.9.1	Heinz et al. (2010)	http://homer.ucsd.edu/; RRID: SCR_010881
deepTools v2.5.4, v3.5.1	Ramírez et al. (2014)	https://deeptools.readthedocs.io/en/develop/; RRID: SCR_016366
Integrative Genomics Viewer (IGV) v2.11.2	Robinson et al. (2011)	https://software.broadinstitute.org/software/igv/; RRID: SCR_011793
csaw v1.20.0, v1.28.0	Lun and Smyth (2015)	https://bioconductor.org/packages/csaw
bedtools v2.27.1, v2.30.0	Quinlan, 2014	https://bedtools.readthedocs.io/; RRID: SCR_006646
GIGGLE	Layer et al. (2018)	http://dbtoolkit.cistrome.org/
edgeR v3.28.1	Robinson et al. (2010)	https://bioconductor.org/packages/edgeR/; RRID: SCR_012802
stageR v1.8.0	Van den Berge et al. (2017)	https://bioconductor.org/packages/release/bioc/html/stageR.html
ReactomePA v1.34.0	Yu and He (2016)	https://www.bioconductor.org/packages/ReactomePA; RRID: SCR_019316
PEGS v0.5.1	Briggs et al. (2021)	https://github.com/fls-bioinformatics-core/pegs
ggplot2	Hadley Wickham	https://ggplot2.tidyverse.org; RRID: SCR_014601
ggpubr	Alboukadel Kassambara	https://rpkgs.datanovia.com/ggpubr/; RRID: SCR_021139

### Resource availability

#### Lead contact

Further information and requests for resources and reagents should be directed to and will be fulfilled by the lead contact, David W Ray (david.ray@ocdem.ox.ac.uk).

#### Materials availability

This study did not generate new unique reagents or materials.

### Experimental model and subject details

#### Animals

Male mice were used throughout, to eliminate sex as a confounder. All animals had *ad libitum* access to standard laboratory chow and water, and were group-housed at room temperature (22°C) on 12hr:12hr light-dark cycles. Experimental mice (i.e. *Cre* transgene carriers) and floxed littermate controls were housed together, randomly assigned to cages at weaning, and then randomly assigned to treatment groups.

#### Hnf4a^fl/fl^Alb^Cre+/-^ mice

Dexamethasone and vehicle treatment of *Hnf4a*^*fl/fl*^*Alb*^*Cre+/-*^ mice (aged 6-8 weeks), generated as described (Hayhurst et al., 2001), was carried out at the National Cancer Institute as previously described (Caratti et al., 2018). The National Cancer Institute Animal Care and Use Committee approved all animal experiments conducted in these experiments.

#### Hnf6^fl/fl^Alb^CreERT2+/−^ mice

*Hnf6*^*fl/fl*^*Alb*^*CreERT2+/−*^ mice were bred at the University of Manchester using the *Onecut1*^*tm1.1Mga*^*/Mmnc Hnf6*^*fl/fl*^ line (Zhang et al., 2009) (sperm obtained from MMRRC; previously backcrossed to C57BL/6) and the *Alb*^*tm1(cre/ERT2)Mtz*^ line (Schuler et al., 2004) (kindly gifted by Drs Pierre Chambon and Daniel Metzger). Recombination was induced with tamoxifen as described (Hunter et al., 2020). Experiments on *Hnf6*^*fl/fl*^*Alb*^*CreERT2+/−*^ mice (aged 10-12 weeks) were conducted at the University of Manchester in accordance with local requirements and with the UK Animals (Scientific Procedures) Act 1986. Procedures were approved by the University of Manchester Animal Welfare and Ethical Review Body (AWERB) and carried out under licence (project licence 70/8558, held by DAB).

### Method details

#### Glucocorticoid administration

For acute treatment with dexamethasone, mice were injected by the intraperitoneal route with water-soluble dexamethasone (D2915 - Sigma-Aldrich) at a dose of 1mg/kg, dissolved in water for injection to a final dexamethasone concentration of 0.2mg/ml. Corresponding vehicle treatment was an equivalent mass of (2-hydroxypropyl)-beta-cyclodextrin (H107 - Sigma-Aldrich) dissolved in water for injection. For studies of GR binding or chromatin accessibility (ChIP-seq and ATAC-seq), tissue was collected after one or two hours; for studies of gene expression (RNA-seq), tissue was collected after two hours.

#### Chromatin immunoprecipitation (ChIP)

##### Tissue processing and chromatin preparation

Chromatin was prepared from flash-frozen liver tissue using the Active Motif ChIP-IT High Sensitivity kit (Active Motif), employing a modified protocol described in (Hunter et al., 2019). All ChIP experiments were conducted with two biological replicates per group, as per ENCODE standards, with replicates handled separately through *in vivo* to *in silico* steps.

##### Immunoprecipitation (IP) and DNA elution

25μg of chromatin (using Nanodrop-measured concentration) was incubated overnight at 4°C with a GR antibody cocktail (ProteinTech 24050-1-AP (lot 00044414) and Cell Signaling D8H2 (lot 2) (2μl of each per IP reaction)). As described (Hunter et al., 2019), to permit direct comparison between samples, and to control for technical variation between ChIP reactions, a spike-in ChIP normalisation strategy was employed (Egan et al., 2016), with 30ng spike-in chromatin (53083, Active Motif) and 2μg spike-in antibody (61686, Active Motif (lot 34216004)) being included in each IP reaction. To obtain sufficient DNA for next-generation sequencing, three IP reactions were carried out for each sample, and then pooled for the pull-down step. Antibody was pulled down using 10μl washed magnetic protein G agarose beads (ReSyn Biosciences). Beads were washed five times with AM1 Wash Buffer (Active Motif) then DNA eluted as per ChIP-IT kit instructions. ChIP-seq DNA was purified with the MinElute PCR Purification kit (Qiagen) (two 10μl elutions per sample).

##### Library preparation

Library preparation and sequencing steps were carried out by the University of Manchester Genomic Technologies Core Facility, using the TruSeq® ChIP library preparation kit (Illumina) and subsequent paired-end sequencing on the Illumina HiSeq 4000 platform.

##### Raw data processing

Raw FASTQ files were quality checked with FastQC software (v0.11.7, Babraham Bioinformatics). Reads were then trimmed with Trimmomatic (v0.38) (Bolger et al., 2014) and aligned to the reference genomes (mouse (mm10) and Drosophila (dm6) as appropriate) with Bowtie2 (v2.3.4.3) (Langmead and Salzberg, 2012). The resulting SAM (Sequence Alignment Map) files were converted to BAM (Binary Alignment Map) files, sorted and indexed with SAMtools (v1.9) (Li et al., 2009). Duplicates were removed with Picard (v2.18.14, Broad Institute). For published ChIP-seq and DNase-seq data, the sratoolkit package (v2.9.2, NCBI) was used to download FASTQ files from the GEO Sequence Read Archive. These were then processed as above.

#### Assay for transposase-accessible chromatin (ATAC)

##### Transposase reaction and library preparation

A nuclei suspension was prepared from frozen liver tissue according to the Omni-ATAC protocol (Corces et al., 2017). ATAC was performed on 80,000 nuclei per sample (n = 2-3 biological replicates per group) using the Active Motif ATAC-seq kit, as per manufacturer's instructions. Libraries were analysed and quantified using TapeStation (Agilent) and the Collibri Library Quantification Kit (Thermo Fisher Scientific), then sequenced on the Illumina HiSeq 4000.

#### Raw data processing

FASTQ files were processed (to BAM, bigWig and peak files) using the PEPATAC pipeline (v0.9.15) (Smith et al., 2021), specifying the following parameters: --single-or-paired paired --genome mm10 --genome-size mm --blacklist mm10.blacklist.bed. PEPATAC incorporates QC steps; read alignment rates across samples ranged from 88-92%, TSS enrichment scores from 11.1 to 15.8; peak counts from 312,022 to 490,828.

#### RNA sequencing (RNA-seq)

##### Sample and library preparation

RNA extraction from liver tissue (n = 3-6 biological replicates per group) was performed using the ReliaPrep RNA Miniprep system (Promega), as per manufacturer's instructions, incorporating a DNase treatment step. Lysing Matrix D tubes (MP Biomedicals) were used to homogenise tissue. 1μg RNA was supplied to the Genomic Technologies Core Facility for library preparation and paired-end sequencing on the Illumina HiSeq 4000 platform, with the TruSeq® Stranded mRNA assay kit (Illumina) employed as per manufacturer's instructions. Demultiplexing (one mismatch allowed) and BCL-to-Fastq conversion was carried out using bcl2fastq software (v2.17.1.14) (Illumina).

##### Raw data processing

FASTQ files were processed by the Core Bioinformatics Facility, employing FastQ Screen (v0.9.2) (Wingett and Andrews, 2018). Trimmomatic (v0.36) (Bolger et al., 2014) was used to remove adapters and poor quality bases. STAR (v2.5.3a) (Dobin et al., 2013) was used to map reads to the mm10 reference genome; counts per gene (exons, GENCODEM16) were then used in differential expression analysis (see below).

#### Genomic data analysis

##### ChIP-seq peak-calling

Peak-calling was performed using MACS2 (v2.1.1.20160309) (Zhang et al., 2008) with settings for narrow peaks and with a q-value cut-off of 0.01 (parameters set as: -f BAMPE -g mm --keep-dup = 1 -q 0.01 --bdg --SPMR --verbose 0).

##### ChIP-seq differential binding (DB) analysis

This was performed with *csaw* (v1.20.0) (Lun and Smyth, 2015, 2016), incorporating spike-in normalisation, as per the code uploaded to Mendeley Data: https://doi.org/10.17632/k8d386ndz6.3. A false discovery rate (FDR) cut-off of 0.05 was used to define regions of differential GR binding.

##### ATAC-seq differential accessibility (DA) analysis

This was performed using *csaw* (v1.28.0), using code adapted from (Reske et al., 2020), uploaded to Mendeley Data: https://doi.org/10.17632/k8d386ndz6.3, employing Loess normalisation. A false discovery rate (FDR) cut-off of 0.05 was used to define regions of differential chromatin accessibility.

##### ChIP-seq and ATAC-seq coverage

deepTools (bamCoverage and computeMatrix commands) (Ramírez et al., 2014) was used to determine read coverage over regions of interest. The computeMatrix command was used in reference-point mode, with the reference point set as the centre of each region.

##### Peak annotation and motif analysis

HOMER (v4.9.1) (Heinz et al., 2010) was used to annotate peak locations (annotatePeaks.pl). HOMER was also used to analyse the underlying DNA sequence motifs in either MACS2-called peaks or *csaw*-defined DB/DA sites. The findMotifsGenome.pl package was used for motif enrichment analysis, with window size set to 200bp (ChIP-seq) or 500bp (ATAC-seq), and the -mask option set. To determine abundance of specific motifs within a set of regions, we used annotatePeaks.pl with the -m and -hist options set; to determine the scores of GRE motifs detected, we used findMotifsGenome.pl with the -find option. To detect instances of motifs genome-wide, we used HOMER's scanMotifGenomeWide.pl package. Throughout, we used the "GRE(NR),IR3/A549-GR-ChIP-Seq(GSE32465)/Homer" matrix as representative of the canonical GRE, the "HNF4a(NR),DR1/HepG2-HNF4a-ChIP-Seq(GSE25021)/Homer" matrix for the HNF4A motif, and "HNF6(Homeobox)/Liver-Hnf6-ChIP-Seq(ERP000394)/Homer" for HNF6; motif matrices are available at http://homer.ucsd.edu/homer/custom.motifs. HOMER motif files specify a log odds detection threshold; this was left unaltered for detection of 'strong' motifs, and reduced by 3 for detection of 'weak' motifs.

##### Distances between peaks (or motifs)

bedtools (Quinlan, 2014) (intersect and window tools) was used to determine overlap between peak sets, or to determine distances between peaks or motifs.

##### Overlap with CistromeDB database

GIGGLE (Layer et al., 2018) (accessed through the CistromeDB Toolkit portal) was used to look for overlap of sites of interest with published datasets (top 1k peaks in each dataset). The tool was set to apply to the mm10 genome, and transcription factor data.

##### Visualisation of ChIP-seq and ATAC-seq data

Visualisations of ChIP-seq and ATAC-seq signal tracks were created with Integrative Genomics Viewer (IGV) (Robinson et al., 2011) and deepTools (Ramírez et al., 2014).

##### Differential gene expression and pathway analysis

Differentially expressed genes were identified with edgeR (v3.28.1) (Chen et al., 2016; Robinson et al., 2010), and detection of a genotype-treatment interaction effect was performed with limma (v3.42.2) voom (Law et al., 2014) and stageR (v1.8.0) (Van den Berge et al., 2017), as per the code uploaded to Mendeley Data: https://doi.org/10.17632/k8d386ndz6.3. Pathway enrichment analyses were performed with ReactomePA (Yu and He, 2016), using *enrichPathway(genes, organism = "mouse", pvalueCutoff = 0.05, pAdjustMethod = "BH", qvalueCutoff = 0.1, maxGSSize = 2000, readable = FALSE)*.

##### Integration of ChIP-seq and RNA-seq data

PEGS (Peak-set Enrichment of Gene-Sets) (Briggs et al., 2021; Yang et al., 2019) was employed to calculate enrichment (hypergeometric test) of genes of interest within specified distances of peak sets. The genome was set to mm10, and distances (bp) specified as 100, 500, 1000, 5000, 10000, 50000, 100000, 500000, 1000000, 5000000.

#### qPCR

RNA was converted to cDNA with the High Capacity RNA-to-cDNA kit (Applied Biosystems). qPCR was performed with PowerUp SYBR Green Master Mix (Thermo Fisher Scientific) using the StepOne Plus (Applied Biosystems) platform. Expression of *Hnf6* (forward primer: GGCAACGTGAGCGGTAGTTT; reverse primer: TTGCTGGGAGTTGTGAATGCT) and *Hnf4a* (forward: AGAAGATTGCCAACATCAC; reverse: GGTCATCCAGAAGGAGTT) was normalised to *Actb* (forward: GGCTGTATTCCCCTCCATCG; reverse: CCAGTTGGTAACAATGCCATGT).

#### Published datasets

The following datasets were downloaded from the GEO Sequence Read Archive: ZT6 liver DNase-seq (SRR1551954) (Sobel et al., 2017), mouse liver H3K27ac ChIP-seq (SRR5054771) (Armour et al., 2017), mouse liver H3K4me1 ChIP-seq (SRR317236, SRR317235) (Shen et al., 2012), mouse liver H3K27me3 ChIP-seq (SRR566941, SRR566942) (Shen et al., 2012), mouse liver HNF4A ChIP-seq (SRR7634103, SRR7634104, SRR7634105, SRR3151870, SRR3151871, SRR3151878, SRR3151879) (Iwafuchi-Doi et al., 2016; Qu et al., 2018), and mouse GR ChIP-seq from macrophages (SRR5182692) (Oh et al., 2017), white adipose tissue (SRR1732507) (Soccio et al., 2015), and mammary gland (SRR3317323) (Shin et al., 2016).

### Quantification and statistical analysis

#### Experimental design and statistics

Sequencing experiments were designed to meet ENCODE minimum requirements (https://www.encodeproject.org/about/experiment-guidelines/) for sample sizes and read depth. N numbers are indicated in figure legends and in the Results section; throughout, n indicates number of individual biological replicates (i.e. number of mice). Experimental animals were compared with littermate controls, and thus inherently randomised to cages at weaning.

Genomic data analysis methods are described above. Significance cut-offs (e.g. for defining a genomic region or gene expression level as significantly different from controls) are indicated in Results, figure legends and in the STAR Methods section. Plots were created with ggplot2. Where simple statistical tests were performed, type of test and statistical details are indicated in the figure legend, with tests conducted using ggpubr, or with GraphPad Prism v8.

## Data Availability

• ChIP-seq, RNA-seq, and ATAC-seq data have been deposited at ArrayExpress (https://www.ebi.ac.uk/arrayexpress/) and are publicly available, at the following accession numbers: ChIP-seq - ArrayExpress: E-MTAB-10224; RNA-seq - ArrayExpress: E-MTAB-10247; ATAC-seq - ArrayExpress: E-MTAB-10266. Accession numbers are also listed in the key resources table. Outputs of peak-calling, differential binding analysis, differential accessibility analysis, differential expression analyses, HOMER motif discovery and GIGGLE analyses have been uploaded to Mendeley Data (URL: https://data.mendeley.com/datasets/k8d386ndz6/3; DOI: https://doi.org/10.17632/k8d386ndz6.3). This paper also analyses existing, publicly available data. The accession numbers for these datasets are listed in the key resources table. • The R code for differential binding, differential accessibility, and differential expression analyses has been deposited at Mendeley Data (URL: https://data.mendeley.com/datasets/k8d386ndz6/3; DOI: https://doi.org/10.17632/k8d386ndz6.3) and is publicly available. • Any additional information required to reanalyse the data reported in this paper is available from the lead contact upon request.

## Supplemental information

Document S1. Figures S1–S4 and Table S3

Table S1. Genes up- and downregulated by dexamethasone treatment in mouse liver (from Caratti et al., 2018), related to Figure 1

Table S2 Genes up- and downregulated by dexamethasone treatment in mouse liver, with and without an effect of *Hnf4a* deletion, related to Figure 5

Document S2. Article plus supplemental information
