# Supplementary material for: HNF4A modulates glucocorticoid action in the liver
Source: Cell Rep. 2022 Apr 19;39(3):110697. doi: 10.1016/j.celrep.2022.110697 (PMC9380254; doi:10.1016/j.celrep.2022.110697)
Supplement: Document S1. Figures S1–S4 and Table S3 [file mmc1.pdf]

Cell Reports, Volume *39*

Supplemental information

HNF4A modulates glucocorticoid action in the liver

A. Louise Hunter, Toryn M. Poolman, Donghwan Kim, Frank J. Gonzalez, David A. Bechtold, Andrew S.I. Loudon, Mudassar Iqbal, and David W. Ray

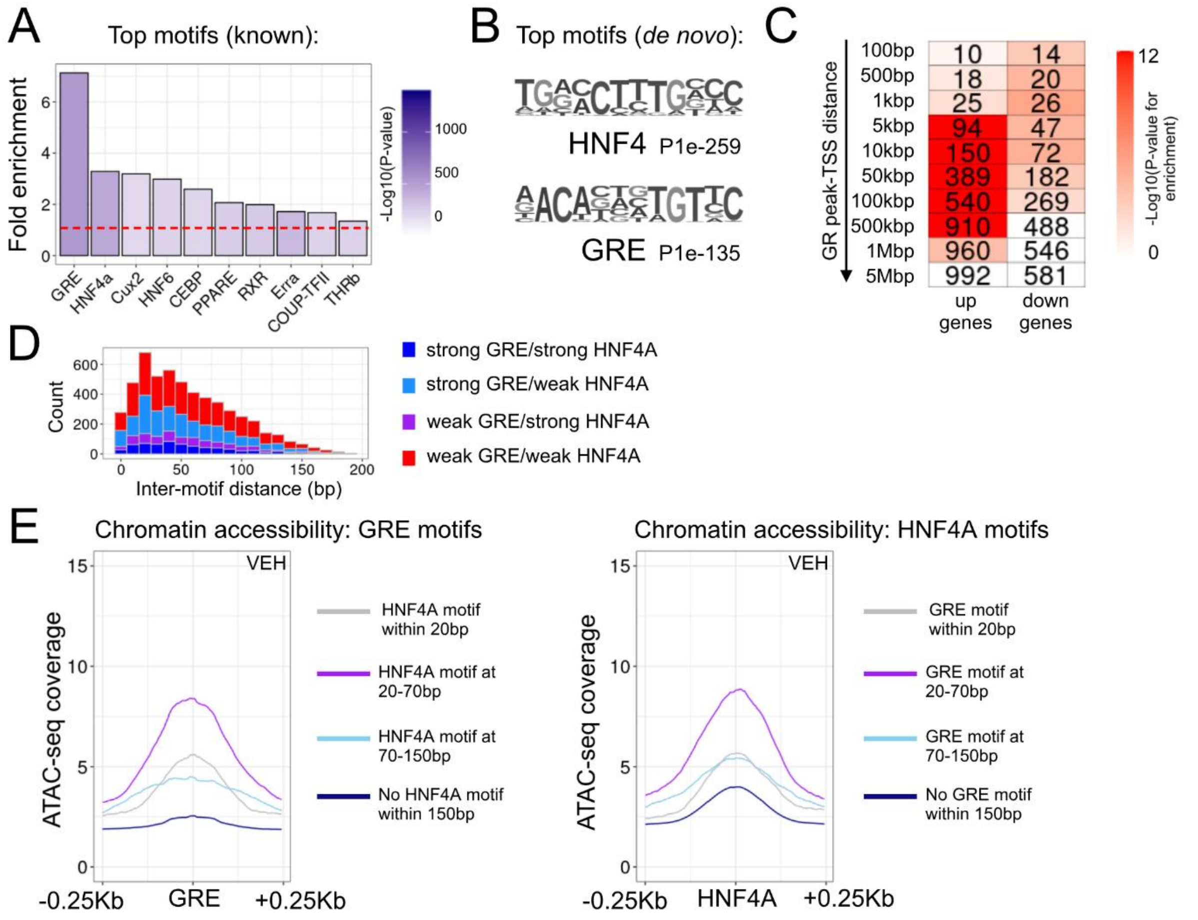

FIGURE S1. Glucocorticoid receptor binding in vehicle-treated liver. Related to Figure 1.

A. Fold enrichment, in GR ChIP-seq peaks from vehicle-treated mouse liver (n=2 biological replicates), of known motifs. Red dotted line at y=1. **B.** The two motifs detected most strongly (lowest P values) *de novo* in GR peaks in vehicle-treated liver. **C.** Heatmap showing enrichment (hypergeometric test) of the transcription start sites (TSSs) of genes up or downregulated by glucocorticoid treatment at increasing distances from GR ChIP-seq peaks (VEH samples). Shading of each cell indicates -log10(P-value) for enrichment (over all genes in the genome), number indicates number of genes in each cluster at that distance. **D.** Histogram of inter-motif distances (10bp bins) for GRE and HNF4A motifs detected within GR ChIP-seq peaks (DEX samples). **E.** ATAC-seq coverage score (mean coverage from 2 biological replicates), in VEH-treated liver, around canonical GRE motifs with or without a HNF4A motif within specified distances (left panel), and around HNF4A motifs with or without a GRE motif within specified distances (right panel).

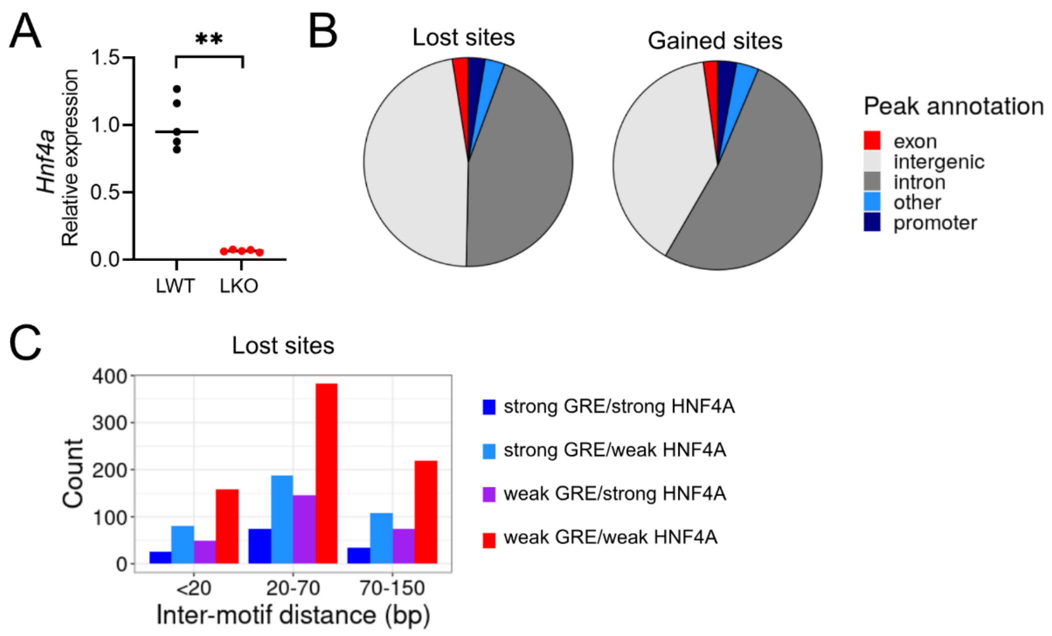

FIGURE S2. GR binding in mouse liver, in presence and absence of *Hnf4a.* Related to Figure 2.

A. Expression of *Hnf4a* (as measured by qPCR, normalised to *Actb* expression) in *Hnf4a*$^{fl/fl}$*Alb*^{Cre} LWT and LKO mouse liver. **P<0.01, Mann Whitney test. Individual data points shown (n=5/group), line at median. **B.** Piecharts showing annotated locations of GR sites lost (left) and gained (right) with *Hnf4a* deletion. **C.** Barchart of inter-motif distances for GRE and HNF4A motifs detected within lost GR sites.

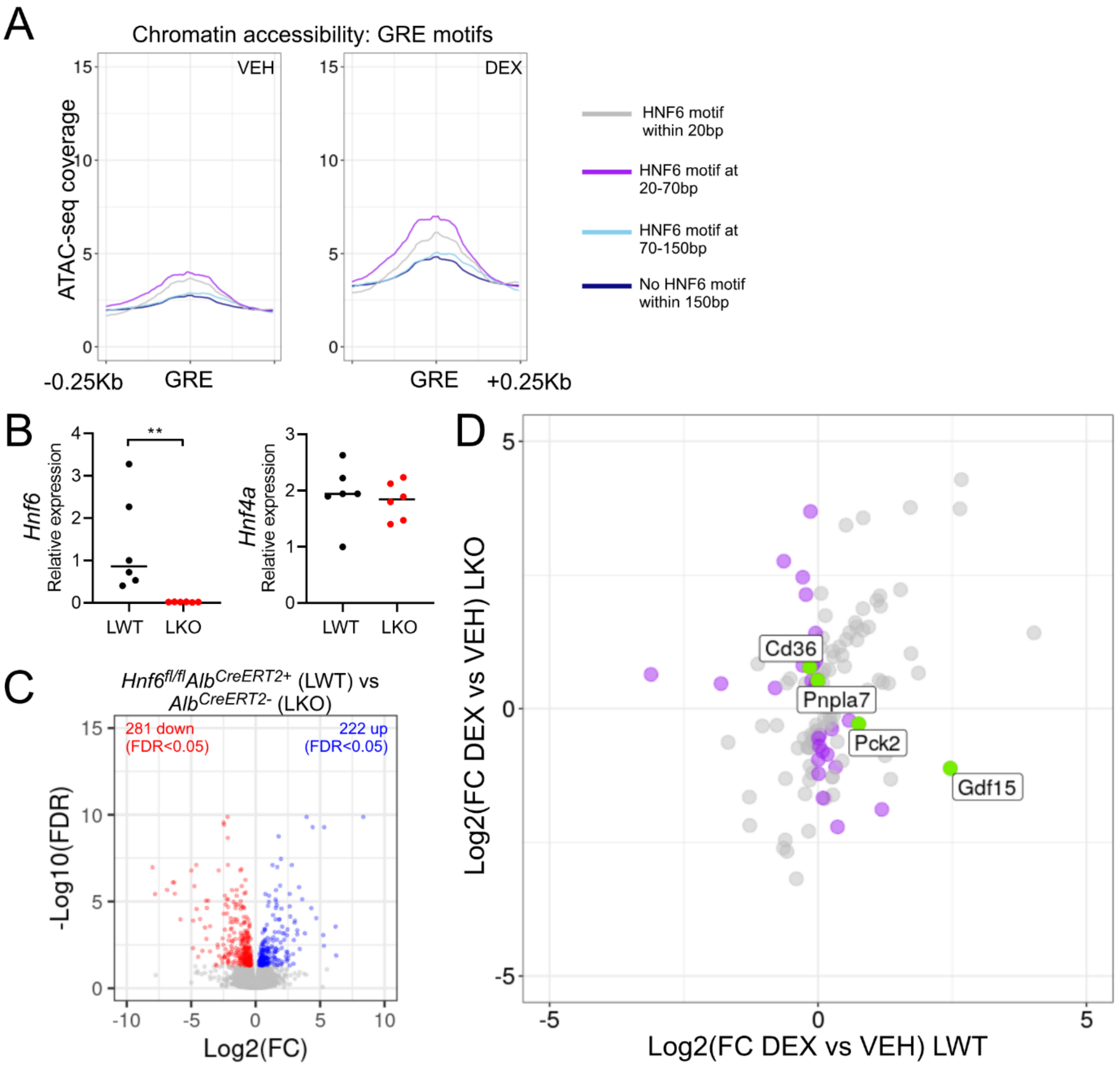

FIGURE S3. Impact of *Hnf6* deletion on glucocorticoid action in mouse liver. Related to Figure 1.

A. ATAC-seq coverage score in VEH-treated liver (left panel, n=2) and DEX-treated liver (right panel, n=3), around canonical GRE motifs with or without a HNF6 motif within specified distances. **B.** Liver expression of *Hnf6* and *Hnf4a* (as determined by qPCR, normalised to *Actb*) in *Hnf6*$^{fl/fl}$*Alb*^{CreERT2} LWT and LKO mice. **P<0.01, Mann Whitney test. Individual data points shown (n=6/group), line at median. **C.** Liver RNA-seq in *Hnf6*$^{fl/fl}$*Alb*^{CreERT2} mice, vehicle-treated LKO vs vehicle-treated LWT samples (n=4-6 biological replicates/group). Significantly downregulated genes (FDR<0.05) in red, significantly upregulated genes in blue. **D.** Effect of DEX treatment in LWT and LKO mice (n=4-6 biological replicates/group). Genes where stageR detects a significant treatment x genotype interaction shown. Those where direction of (significant) change is different between genotypes highlighted in purple. These include metabolic regulators and enzymes of interest, highlighted in green. FC = fold change.

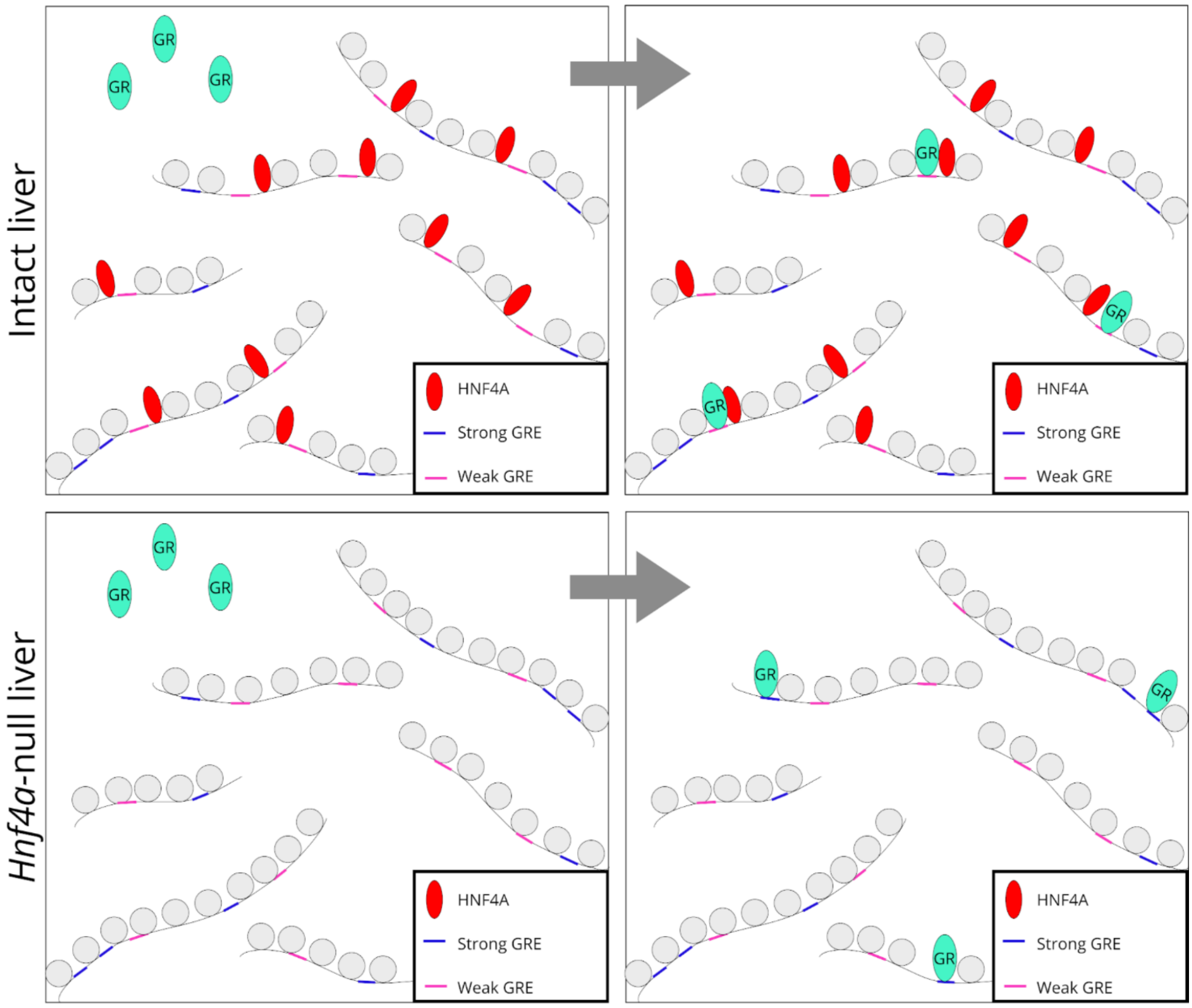

FIGURE S4. Cartoon of proposed patterns of GR binding in intact (*Hnf4a$^{fl/fl}$Alb^{Cre}* LWT) and *Hnf4a*-null (*Hnf4a$^{fl/fl}$Alb^{Cre}* LKO) mouse liver in the course of glucocorticoid treatment. Related to Figures 1 and 3.

In intact liver, HNF4A binding marks sites where open chromatin favours GR binding, even though GREs may show considerable degeneracy from the canonical motif ("Weak GREs"). In *Hnf4a*-null liver, greater similarity to the canonical GRE ("Strong GRES") favours GR binding, as HNF4A-mediated chromatin accessibility is lost.

**A. Genes up-regulated by DEX, unaffected by *Hnf4a* deletion**	**B. Genes up-regulated by DEX, affected by *Hnf4a* deletion**	**C. Genes down-regulated by DEX, unaffected by *Hnf4a* deletion**	**D. Genes down-regulated by DEX, affected by *Hnf4a* deletion**
Common Pathway of Fibrin Clot Formation **Padj 1.91E-06**	Cholesterol biosynthesis **Padj 2.62e-07**	No enriched terms found.	No enriched terms found.
Regulation of TLR by endogenous ligand **Padj 7.35e-06**	Metabolism of steroids **Padj 4.54e-05**		
Formation of Fibrin Clot (Clotting Cascade) **Padj 9.22e-05**	TRAF6 mediated induction of NFkB and MAP kinases upon TLR7/8 or 9 activation **Padj 3.48E-04**		
	MyD88 dependent cascade initiated on endosome **Padj 3.78e-04**		

Table S3 – ReactomePA pathway analysis of genes up- and down-regulated by dexamethasone treatment in mouse liver, with and without an effect of *Hnf4a* deletion. Related to Figure 5.
